# Rare Variants of Putative Candidate Genes Associated With Sporadic Meniere's Disease in East Asian Population

**DOI:** 10.3389/fneur.2019.01424

**Published:** 2020-01-22

**Authors:** Eun Hye Oh, Jin-Hong Shin, Hyang-Sook Kim, Jae Wook Cho, Seo Young Choi, Kwang-Dong Choi, Je-Keun Rhee, Seowhang Lee, Changwook Lee, Jae-Hwan Choi

**Affiliations:** ^1^Department of Neurology, Research Institute for Convergence of Biomedical Science and Technology, Pusan National University School of Medicine, Pusan National University Yangsan Hospital, Yangsan, South Korea; ^2^Department of Neurology, Pusan National University Hospital, Pusan National University School of Medicine and Biomedical Research Institute, Busan, South Korea; ^3^School of Systems Biomedical Science, Soongsil University, Seoul, South Korea; ^4^Department of Biological Sciences, School of Life Sciences, Ulsan National Institute of Sciences and Technology, Ulsan, South Korea

**Keywords:** Meniere's disease, endolymphatic hydrops, gene, whole-exome sequencing, rare variant

## Abstract

**Objectives:** The cause of Meniere's disease (MD) is unclear but likely involves genetic and environmental factors. The aim of this study was to investigate the genetic basis underlying MD by screening putative candidate genes for MD.

**Methods:** Sixty-eight patients who met the diagnostic criteria for MD of the Barany Society were included. We performed targeted gene sequencing using next generation sequencing (NGS) panel composed of 45 MD-associated genes. We identified the rare variants causing non-synonymous amino acid changes, stop codons, and insertions/deletions in the coding regions, and excluded the common variants with minor allele frequency >0.01 in public databases. The pathogenicity of the identified variants was analyzed by various predictive tools and protein structural modeling.

**Results:** The average read depth for the targeted regions was 1446.3-fold, and 99.4% of the targeted regions were covered by 20 or more reads, achieving the high quality of the sequencing. After variant filtering, annotation, and interpretation, we identified a total of 15 rare heterozygous variants in 12 (17.6%) sporadic patients. Among them, four variants were detected in familial MD genes (*DTNA, FAM136A, DPT*), and the remaining 11 in MD-associated genes (*PTPN22, NFKB1, CXCL10, TLR2, MTHFR, SLC44A2, NOS3, NOTCH2*). Three patients had the variants in two or more genes. All variants were not detected in our healthy controls (*n* = 100). No significant differences were observed between patients with and without a genetic variant in terms of sex, mean age of onset, bilaterality, the type of MD, and hearing threshold at diagnosis.

**Conclusions:** Our study identified rare variants of putative candidate genes in some of MD patients. The genes were related to the formation of inner ear structures, the immune-associated process, or systemic hemostasis derangement, suggesting the multiple genetic predispositions in the development of MD.

## Introduction

Meniere's disease (MD) is a clinical syndrome that consists of episodes of spontaneous vertigo usually associated with unilateral fluctuating sensorineural hearing loss (SNHL), tinnitus and aural fullness (1). Almost 150 years have elapsed since Prosper Meniere first described the clinical entity, but unfortunately, the exact pathophysiology of this condition is yet to be fully understood. Histopathological studies in human temporal bones have found endolymphatic hydrops (EH) in most patients with MD, but the origin of the EH is unknown (2).

MD is considered a multifactorial disorder associated with the various factors including anatomical abnormalities, infections, allergens, and autoimmune disorders. Most MD cases are sporadic, but familial MD is observed in 8–10% of sporadic cases, suggesting genetic susceptibility to the development of MD (3–5). To search a candidate gene associated with MD, many approaches including linkage analysis, case-control study or the sequencing of selected genes have been previously used (6–10). A variety of common single nucleotide polymorphisms (SNPs) have been meaningfully detected in patients with MD (11–35) (Supplementary Table 1). Most of them were the genes related to the inflammation or regulating the ionic composition and water transport of the inner ear. However, the effect size of these common variants was small (odds ratio < 2). Furthermore, a common variant could not reflect the entire heritability of MD. Recent studies using whole-exome sequencing (WES) for Spanish families with MD have identified probably pathogenic rare variants in candidate genes including *FAM136A, DTNA, PRKCB, DPT*, and *SEMA3D* (36–38). Since these genes encode proteins that may be relevant to the formation or maintaining of inner ear structures, the identified rare variants are expected to account for the genetic contribution of MD, but further replicative studies in distinct populations are needed. Thus, the aim of this study was to explore the previously proposed MD-associated genes using targeted NGS to investigate the genetic basis underlying MD.

## Materials and Methods

### Subjects

We recruited 68 unrelated patients with definite MD who visited a tertiary dizziness clinic from 2015 to 2018. The diagnosis of definite MD was made based on the criteria established by the Classification Committee of the Barany Society (1). All patients met the following criteria: (1) Two or more spontaneous episodes of vertigo, each lasting 20 min to 12 h, (2) Audiometrically documented low- to medium-frequency sensorineural hearing loss in the affected ear on at least one occasion before, during, or after one of the episodes of vertigo, (3) Fluctuating aural symptoms (hearing, tinnitus, or earfullness) in the affected ear, (4) Not better accounted for by another vestibular diagnosis. A brain MRI was performed to rule out any neurological lesions. The patients included 38 males and 30 females with age ranging from 28 to 89 years (mean age 60.2 ± 12.0 years). The mean age of onset was 57.5 ± 11.3 years. Most patients (*n* = 63, 93%) had a unilateral MD. Six had at least one family member with a history of MD-like symptoms. According to the phenotype, 18 (26%) were classified as delayed MD based on a previous history of sensorineural hearing loss (months or years) before the onset of vertigo episodes, while the others (*n* = 50) showed classic MD phenotype (39).

All experiments followed the tenets of the Declaration of Helsinki, and informed consents were obtained after the nature and possible consequences of this study had been explained to the participants. This study was approved by the institutional review boards of Pusan National University Yangsan Hospital.

### Targeted Next-Generation Sequencing

Targeted genes were collected from the literature review. The keywords “Meniere's disease” and “gene” were used to search the MD-associated genes in PubMed, resulting in 101 papers when this study was initiated (August, 2017). After excluding the genes showing no correlation with MD, we selected 45 genes used for targeted NGS (Supplementary Table 1). The selected genes were largely classified into two categories as follow: (1) “familial MD gene,” the pathogenic genes for familial MD identified by high-throughput sequencing (36–38); (2) “MD-associated gene,” the candidate genes contributing to the development of MD demonstrated by association study or network-based study (11–35, 40). The MD gene panel was designed by the Suredesign webtool (Agilent) to cover the exons and 20 bp in the flanking regions.

Genomic DNA was extracted from the blood sample of all patients. For the generation of standard exome capture libraries, we used the Agilent SureSelect Target Enrichment protocol for Illumina paired-end sequencing library (ver. B.3, June 2015) with 1 μg input DNA. The quantification of DNA and the DNA quality was measured by PicoGreen and Nanodrop. The qualified genomic DNA sample was randomly fragmented by Covaris followed by adapter ligation, purification, hybridization, and PCR. Captured libraries were subjected to Agilent 2100 Bioanalyzer to estimate the quality and were loaded on to the Illumina HiSeq2500 (San Diego, USA) according to the manufacturer's instructions. Raw image files were processed by HCS1.4.8 for base-calling with default parameters and the sequences of each individual were generated as 100 bp paired-end reads. Sequence reads were aligned to the human reference genome sequence (GRCh37.3, hg19) using the Burrows-Wheeler Aligner (BWA, version 0.7.12). PCR duplicate reads were marked and removed with Picard tools (version 1.92). Genome Analysis Toolkit (GATK, version 2.3-9) was used for indel realignment and base recalibration. Variation annotation and interpretation analysis were performed using SnpEff (version 4.2).

### Identification of Rare or Novel Variants

To identify the possible pathogenic variants, we first filtered out synonymous and non-coding variants, and extracted the variants causing non-synonymous amino acid changes, stop codons, in-frame insertions/deletions in coding regions, or changes to splice site sequences in exon/intron boundaries. Then, common variants with a minor allele frequency (MAF) > 0.01 that represented in dbSNP147, the Exome Aggregation Consortium (ExAC), gnomAD, 1,000 Genomes Project, and NHLBI GO Exome Sequencing Project (ESP) 6500 were excluded. The pathogenicity of the non-synonymous variants was analyzed using Sorting Intolerant From Tolerant (SIFT), Likelihood Ratio Test (LRT), Polyphen2, MutationTaster, Functional Analysis Through Hidden Markov Models (FATHMM), and Combined Annotation Dependent Depletion (CADD) phred score. The higher CADD phred scores indicate that a variant is more likely to have deleterious effects (41). The strength of ectopic splicing sites created by intronic variants was evaluated by the Human Splicing Finder program. All rare and novel variants were annotated for previously reported disease-causing variants using the Human Gene Mutation Database. All variants were confirmed by Sanger DNA sequencing, and were screened in 100 normal controls.

### Protein Structural Modeling

The structural modeling of missense variants of *PTPN22* (PDB accession code: 2P6X), *MTHFR* (PDB accession code: 6FCX), *NOTCH2* (PDB accession code: 2OO4) and *CXCL10* (PDB accession code: 1O80) were generated using I-TASSER server. The confidence scores (C-scores) indicating significance of threading template alignments and the convergence parameters of modeling simulation were calculated for each predicted model to estimate the quality of model (42). The calculated C-scores of *PTPN22, MTHFR, NOTCH2, CXCL10* were 1.61, 0.07, 0.36, 0.34, respectively. The best model was selected based on the template modeling score (TM-score) and the root mean square deviation (RMSD) value indicating proper topology of the mutant model compared to the PDB template. The optimization of predicted models was performed by PyMOL (The PyMOL Molecular Graphics System, Version 2.0 Schrödinger, LLC.), and each mutant model was aligned with that of wild type to compare three-dimensional structures.

### Statistical Analyses

To investigate the association between the genetic variants and disease manifestation, we calculated the odds ratio for each variant using Fisher's exact test from the allele frequency of ExAC and gnomAD database as controls (total population and East Asian population). *P*-values were corrected for multiple testing by the total amount of variants found for each gene following Bonferroni approach. We also compared the clinical characteristics between patients with and without rare variants. Continuous variables (mean age, age of onset, and hearing threshold at diagnosis) were compared using the Mann-Whitney test, and categorical variables (sex, bilaterality, the type of MD, and the presence of family history) using Fisher's exact test. A statistical analysis was performed using SPSS software (SPSS Inc., Chicago, IL, USA) and a *p* < 0.05 was considered as significant.

## Results

The average read depth for the targeted regions was 1446.3-fold, and 99.4% of the targeted regions were covered by 20 or more reads, achieving the high quality of the sequencing (Supplementary Table 2). After variant filtering, annotation, and interpretation, we identified a total of 15 rare heterozygous variants (two novel and 13 rare variants) in 12 (17.6%) sporadic patients (Table 1). Among them, four variants were detected in familial MD gene, and the remaining 11 in MD-associated gene. Three patients (P-29, P-60, P-66) had the variants in two or more genes. All variants were not detected in our healthy controls (*n* = 100).

**Table 1:** Identified rare variants in putative candidate genes associated with Meniere's disease.

**Gene**	**mRNA**	**Protein**	**Variant effect**	**Patient's ID**	**dbSNP**	**ExAC MAF**	**gnomAD MAF**	***In silico*** **prediction**					**CADD** **phred score**
								**SIFT**	**Polyphen**	**LRT**	**Mutation taster**	**FATHMM**	
**Familial MD genes**													
*DTNA*	c.1002-4A > G	(–)	Aberrant splicing	P-31	rs369005625	0.00018	0.00025	(–)	(–)	(–)	(–)	(–)	21.8
*DTNA*	c.2094G > A	p.Trp698^*^	Nonsense	P-66	(–)	0	0	(–)	(–)	U	D	(–)	44
*FAM136A*	c.238G > T	p.Ala80Ser	Missense	P-29	rs199565792	0.00009	0.00005	T	B	N	N	(–)	18.63
*DPT*	c.16C > T	p.Leu6Phe	Missense	P-13	rs192608693	0.00005	0.00003	T	P	D	D	T	16.52
**MD-associated genes**													
*PTPN22*	c.205A > G	p.Ser69Gly	Missense	P-26	rs202095629	<0.00001	0	D	D	D	D	D	26.2
*PTPN22*	c.829G > T	p.Glu277^*^	Nonsense	P-60	rs72483511	<0.00001	0.00003	(–)	(–)	D	D	(–)	37
*PTPN22*	c.1356delT	p.Phe452Leufs^*^3	Deletion	P-65	(–)	0	0	(–)	(–)	(–)	(–)	(–)	(–)
*NFKB1*	c.1799A > C	p.Glu600Ala	Missense	P-49	rs55661548	0.00031	0.00045	T	B	N	D	T	22.5
*TLR2*	c.1339C > T	p.Arg447^*^	Nonsense	P-54	rs62323857	0.00037	0.00029	(–)	(–)	N	D	(–)	35
*CXCL10*	c.85C > T	p.Arg29Cys	Missense	P-29	rs11548618	0.00611	0.00608	D	D	D	D	T	25.3
*MTHFR*	c.136C > T	p.Arg46Trp	Missense	P-5/P-35	rs138189536	0.00022	0.00020	T	D	D	D	D	25.3
*MTHFR*	c.742A > G	p.Ile248Val	Missense	P-66	(–)	0.00002	0.00003	D	D	N	D	D	22.5
*SLC44A2*	c.761G > A	p.Arg254His	Missense	P-29	(–)	0.00002	0.00003	D	D	D	D	T	31
*NOS3*	c.2207G > A	p.Arg736Gln	Missense	P-60	rs544887797	0.00008	0.00010	D	D	D	D	T	33
*NOTCH2*	c.4688G > A	p.Arg1563His	Missense	P-63	rs76770652	0.00002	0.00002	T	B	U	D	D	22.7

*MAF, minor allele frequency; MD, Meniere's disease*.

*Transcript ID: DTNA, NM_001198938.1; FAM136A, NM_032822.2; DPT, NM_001937.5; PTPN22, NM_015967.7; NFKB1, NM_003998.4; TLR2, NM_003264.4; CXCL10, NM_001565.4; MTHFR, NM_005957.4; SLC44A2, NM_020428.4; NOS3, NM_000603.5; NOTCH2, NM_024408.4*.

*SIFT- D (damaging), T (tolerated); Polyphen- D (probably damaging), P (possibly damaging), B (benign); LRT- D (deleterious), N (neutral), U (unknown); MutationTaster- D (disease_causing), N (polymorphism); FATHMM- D (deleterious), T (tolerated)*.

### Variants in Familial MD Genes

**Figure 1:**
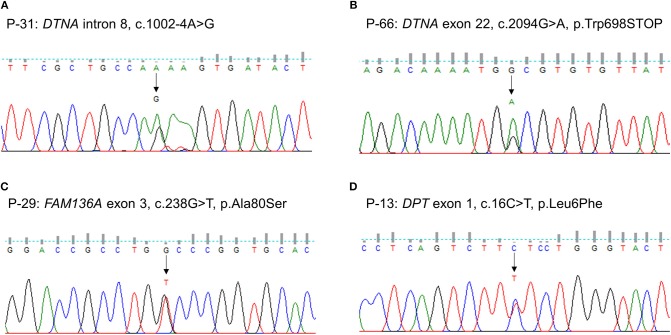
Sequencing results of the rare variants in familial MD genes identified by targeted next-generation sequencing. The chromatograms show two rare heterozygous variants in *DTNA*: **(A)** one is a splice site variant at the 4th nucleotide of the splice acceptor site in intron 8 (c.1002-4A > G, P-31); **(B)** another is a novel nonsense variant in exon 22 causing a premature stop codon (c.2094G > A, p.W698X, P-66). The others are a heterozygous missense rare variant in exon 3 of *FAM136A* causing the substitution of alanine by serine at position 80 (c.238G > T, p.A80S, P-29) **(C)** and in exon 1 of *DPT* changing the highly conserved leucine by phenylalanine at position 6 (c.16C > T, p.L6F, P-13) **(D)**.

We identified one novel and three rare heterozygous variants in three familial MD genes. Two variants were detected in *DTNA*. One was a rare variant affecting splice acceptor site located in intron 8 (P-31: c.1002-4A > G, Figure 1A). Human Splicing Finder predicted that this variant will alter the splice site and make new splice sites resulting in three bases longer on exon 9. Another was a nonsense variant resulting in a premature stop codon (P-66: c.2094G > A, p.W698X, Figure 1B). It was absent in all public database, and predicted to be deleterious by MutationTaster and CADD scores. The other rare variants were detected in *FAM136A* (P-29: c.238G > T, p.A80S, Figure 1C) and *DPT* (P-13: c.16C > T, p.L6F, Figure 1D). Both were a missense variant which a single nucleotide change results in a codon that codes for a different amino acid. Of them, the variant in *DPT* changed highly conserved amino acid and was predicted as protein-damaging potential by three prediction tools (Polyphen2, LRT, MutationTaster).

### Variants in MD-Associated Genes

**Figure 2:**
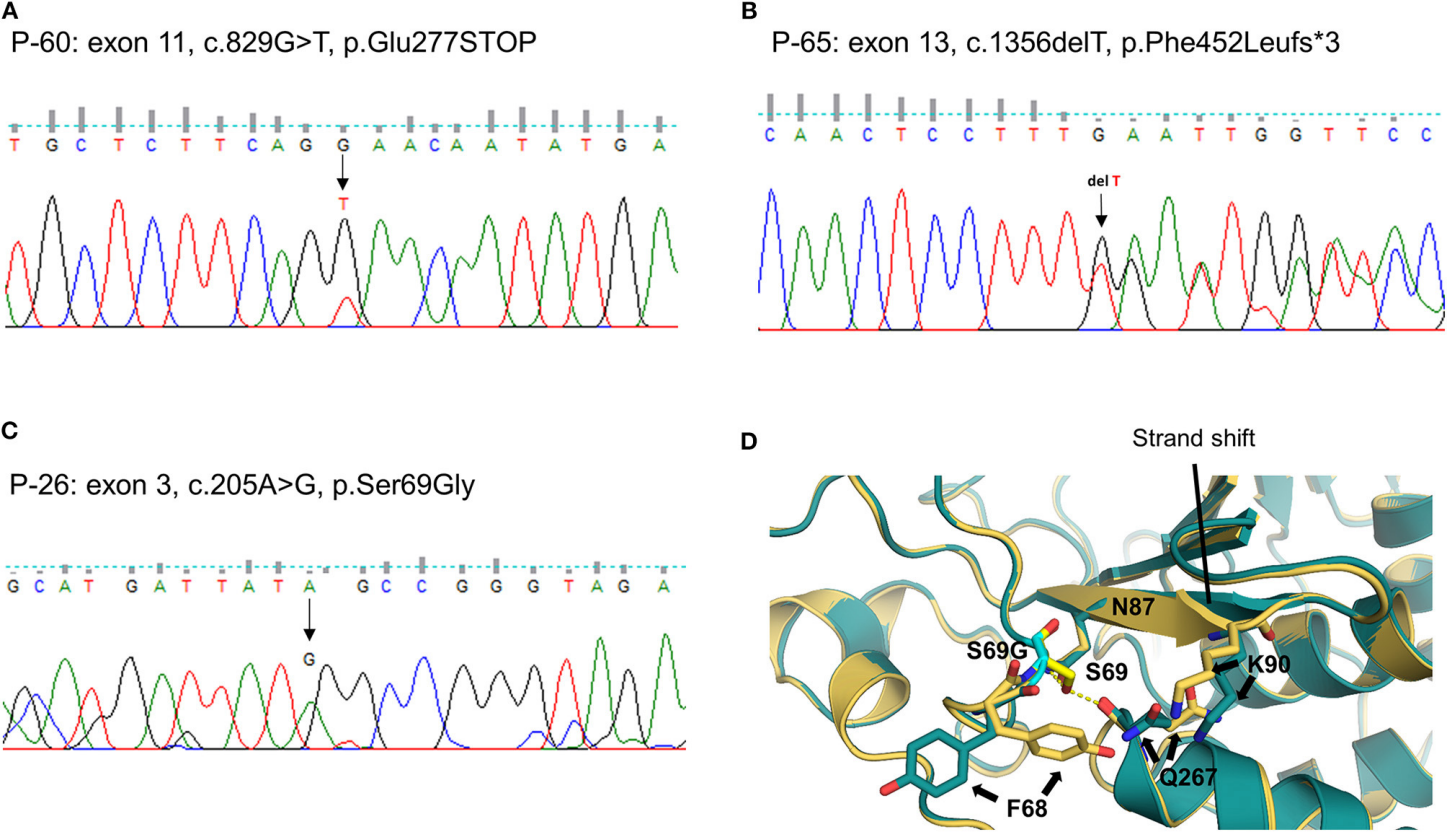
Sequencing results and protein structural modeling of the rare variants in *PTPN22* identified by targeted next-generation sequencing. The chromatograms show three rare heterozygous variants: **(A)** one is a nonsense variant in exon 11 resulting in a premature stop codon (c.829G > T, p.E277X, P-60); **(B)** another is single base deletion in exon 13 that leads to frameshift and premature stop codon 3 amino acids downstream (c.1356delT, p.F452Lfs*3, P-65); **(C)** the other is a missense rare variant in exon 3 causing the substitution of highly conserved serine by glycine at position 69 (c.205A > G, p.S69G, P-26). Protein structural modeling predicts that p.S69G variant may affect the enzyme activity of PTPN22 by a disruption of hydrogen bonds network between p.S69 and p.N87 residues, and the steric hindrance effect resulting from the rotation of p.F68, p.Q267, and p.K90 residues **(D)**, yellow: wild type PTPN22, teal: p.S69G mutant PTPN22).

We identified one novel and 10 rare heterozygous variants in eight MD-associated genes. Interestingly, possibly pathogenic variants of *PTPN22* were detected in three unrelated patients. Two were truncated variants caused by premature stop codon (P-60: c.829G > T, p.E277X, Figure 2A) or single base deletion (P-65: c.1356delT, p.F452Lfs^*^3, Figure 2B). The nonsense variant was predicted to be deleterious by LRT, MutationTaster and CADD score. The remaining one was a missense variant with protein-damaging potential predicted by all prediction tools (P-26: c.205A > G, p.S69G, Figure 2C). Protein structural modeling predicted that p.S69G variant may disrupt the positions of surrounding residues (p.F68, p.N87, p.K90, and p.Q267) by steric hindrance effects, and eventually may affect the enzymatic activity of PTPN22 (Figure 2D).

**Figure 3:**
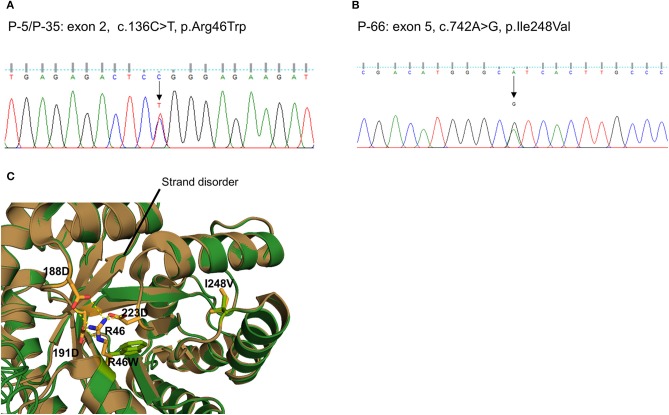
Sequencing results and protein structural modeling of the rare variants in *MTHFR* identified by targeted next-generation sequencing. The chromatograms show two rare heterozygous variants: **(A)** one is a missense rare variant in exon 2 causing the substitution of highly conserved arginine by tryptophan at position 46 (c.136C > T, p.R46W, P-5/P-35); **(B)** another is also a missense rare variant in exon 5 causing the substitution of highly conserved isoleucine by valine at position 248 (c.742A > G, p.I248V, P-66). **(C)** Protein structural modeling shows that p.R46W variant may cause breakage of salt bridges with side chains of p.D188, p.D223, and p.D191 residues. This leads to destabilize the central β-sheet which is essential for reductase activity. On the other hands, p.I248V variant reveals no discernible differences when secondary structure as well as surrounding residue orientation are analyzed (brown: wild type MTHFR, green: p.R46W/p.I248V mutant MTHFR).

We also detected two missense variants of *MTHFR* in three unrelated patients (P-5 and P-35: c.136C > T, p.R46W, Figure 3A; P-66: c.742A > G, p.I248V, Figure 3B). They were considered likely pathogenic by four of the prediction tools. Protein structural modeling revealed that p.R46W variant may cause breakage of salt bridges with surrounding residues (p.D188, p.D191, and p.D223), resulting in destabilizing the β-strand (β5and β6 Figure 3C). On the other hand, p.I248V variant showed no discernible differences when the secondary structure as well as surrounding residue orientation were analyzed.

**Figure 4:**
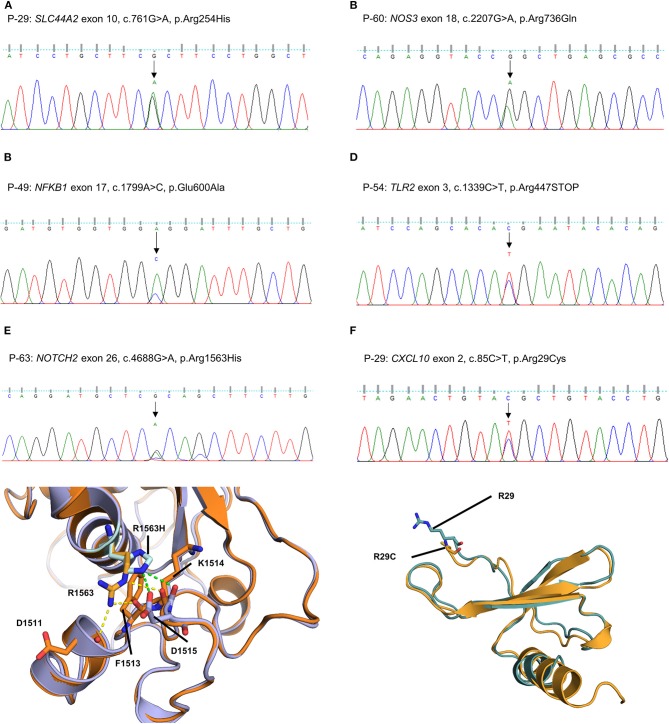
Sequencing results and protein structural modeling of the rare variants in other MD-associated genes identified by targeted next-generation sequencing. **(A–D)** The chromatograms show a heterozygous missense rare variant in *SLC44A2* (exon 10, c.761G > A, p.R254H, P-29), *NOS3* (exon 18, c.2207G > A, p.R736Q, P-60), and *NFKB1* (exon 17, c.1799A > C, p.E600A, P-49), and a nonsense variant in exon 3 of *TLR2* causing premature stop codon (c.1339C > T, p.R447X, P-54). **(E)** P-63 has a heterozygous missense variant in exon 26 of *NOTCH2* causing the substitution of highly conserved arginine by histidine at position 1563 (c.4688G > A, p.R1563H). In protein structural modeling, p.R1563H variant may result in loss of interaction with p.D1511 and p.F1513 residues, and form new hydrogen bond network with main chain of p.K1514 residue and side chain of p.D1515 (green dash line). These impaired interactions make p.D1515 residue to lose ability to interact with a calcium ion, and eventually destabilize the Lin-12/Notch repeat domain required for regulating the NOTCH2 receptor (orange: wild type NOTCH2, purple: p.R1563H mutant NOTCH2). **(F)** P-29 had a heterozygous missense rare variant in exon 2 of *CXCL10* causing the substitution of highly conserved arginine by cystein at position 85 (c.85C > T, p.R29C). However, there is no conformational change in overall structure because the R29 residue is located at the N-terminal end and not involved in interaction with neighboring residues (teal: wild type CXCL10, orange: p.R29C mutant CXCL10).

The other five missense variants had protein-damaging potential predicted by at least one prediction tool: *SLC44A2* (P-29: c.761G > A, p.R254H, Figure 4A), *NOS3* (P-60: c.2207G > A, p.R736Q, Figure 4B), *NFKB1* (P-49: c.1799A > C, p.E600A, Figure 4C), *NOTCH2* (P-63: c.4688G > A, p.R1563H, Figure 4E), and *CXCL10* (P-29: c.85C > T, p.R29C, Figure 4F). Protein structural modeling predicted that the p.R1563H variant in *NOTCH2* may destabilize the Lin-12/Notch repeat (LNR) domain by impairing a hydrogen bond network with surrounding residues (p.D1511, p.F1513, and p.D1515), while the p.R29C variant in *CXCL10* may cause no conformational change in overall structure. The remaining one was a nonsense variant of *TLR2* (P-54: c.1339C > T, p.R447X, Figure 4D), which was predicted to be deleterious by MutationTaster and CADD score.

Copy number variation (CNV) analysis using CNVkit from targeted DNA sequencing data did not reveal any pathogenic CNV in each patient.

### Association Analysis of a Genetic Variant

When using the allele frequency of total population from ExAC and gnomAD database, 14 of the 15 rare variants were significantly associated with MD (unadjusted *p*-value), but eight of them showed significant association after correcting for multiple testing (Bonferroni) in both databases (Table 2). The genes included *DTNA, PTPN22, MTHFR, SLC44A2*, and *NOTCH2*. In particular, all rare variants of *PTPN22* and *MTHFR* were significantly associated with MD even after correcting for multiple testing. Compared to the allele frequency of East Asians, 10 variants were significantly associated with MD (unadjusted *p*-value) in both databases, but no significant associations were observed in all variants after correcting for multiple testing (Table 2).

**Table 2:** Genetic association analysis results for rare variants.

**Gene**	**Variant**	**Frequency** **of variant** **in this study**	**ExAC**						**gnomAD**					
			**Odd ratio, total^a^**	***p*-value**	**Adjusted *p*-value^b^**	**Odd ratio, East asian^a^**	***p*-value**	**Adjusted *p*-value^b^**	**Odd ratio, total^a^**	***p*-value**	**Adjusted *p*-value^b^**	**Odd ratio, East asian^a^**	***p*–value**	**Adjusted *p*–value^b^**
*DTNA*	c.1002-4A > G	1/68	119.24 (1.5–42.4)	**0.0129**	0.1930	8.50 (0.6–18.9)	0.1654	1.0000	87.04 (1.3–36.5)	**0.0174**	0.2610	6.94 (0.4–12.2)	0.1977	1.0000
*DTNA*	c.2094G > A	1/68	5396.11 (1.7–1034.5)	**0.0006**	**0.0084**	384.64 (0.5–328.5)	**0.0077**	0.1155	5345.89 (1.7–1030.3)	**0.0006**	**0.0090**	442.78 (0.6–349.3)	**0.0067**	0.1005
*FAM136A*	c.238G > T	1/68	233.12 (1.9–59.5)	**0.0067**	0.1010	16.69 (0.6–18.9)	0.0897	1.0000	373.78 (2.2–78.6)	**0.0043**	0.0645	30.90 (0.7–26.6)	0.0511	0.7665
*DPT*	c.16C > T	1/68	376.81 (2.2–78.8)	**0.0043**	0.0646	26.88 (0.7–25.1)	0.0584	0.8760	692.25 (2.5–117.7)	**0.0024**	**0.0360**	57.22 (0.8–39.9)	**0.0296**	0.4440
*PTPN22*	c.205A > G	1/68	1707.67 (2.6–246.7)	**0.0012**	**0.0177**	125.83 (0.9–79.5)	**0.0158**	0.2370	5345.89 (1.7–1030.3)	**0.0005**	**0.0075**	442.78 (0.6–349.3)	**0.0067**	0.1005
*PTPN22*	c.829G > T	1/68	1791.13 (2.7–251.9)	**0.0011**	**0.0169**	127.99 (0.8–80.1)	**0.0155**	0.2325	684.10 (2.5–117.1)	**0.0025**	**0.0375**	80.36 (0.9–51.6)	**0.0222**	0.3330
*PTPN22*	c.1356delT	1/68	5396.11 (1.7–1034.5)	**0.0006**	**0.0084**	384.64 (0.5–328.5)	**0.0077**	0.1155	5345.89 (1.7–1030.3)	**0.0006**	**0.0090**	442.78 (0.6–349.3)	**0.0067**	0.1005
*NFKB1*	c.1799A > C	1/68	70.06 (1.2–32.9)	**0.0216**	0.3241	5.11 (0.4–10.1)	0.2577	1.0000	49.02 (1.1–27.9)	**0.0306**	0.4590	4.19 (0.4–9.6)	**0.0141**	0.2115
*TLR2*	c.1339C > T	1/68	59.18 (1.1–30.5)	**0.0255**	0.3821	54.92 (0.8–39.2)	**0.0308**	0.4620	75.26 (1.3–34.0)	**0.0201**	0.3015	49.18 (0.8–35.0)	**0.0334**	0.5010
*CXCL10*	c.85C > T	1/68	3.59 (0.3–8.8)	0.3427	1.0000	126.87 (0.8–79.8)	**0.0156**	0.2340	3.63 (0.3–8.8)	0.3396	1.0000	141.36 (0.9–83.6)	**0.0141**	0.2115
*MTHFR*	c.136C > T	2/68	163.70 (2.5–34.2)	**0.0001**	**0.0019**	13.79 (0.8–11.8)	**0.0160**	0.0019	184.44 (2.6–36.2)	**0.0001**	**0.0015**	19.17 (0.9–13.8)	**0.0087**	0.1305
*MTHFR*	c.742A > G	1/68	770.26 (2.6-123.3)	**0.0022**	**0.0336**	54.88 (0.8-39.1)	**0.0308**	0.2400	593.97 (2.5-103.2)	**0.0028**	**0.0420**	49.17 (0.8–35.0)	**0.0334**	0.5010
*SLC44A2*	c.761G > A	1/68	1079.20 (2.7–159.4)	**0.0017**	**0.0252**	76.91 (0.9–50.6)	**0.0232**	0.3480	694.64 (2.5–117.8)	**0.0025**	**0.0300**	80.40 (0.9–51.6)	**0.0222**	0.3330
*NOS3*	c.2207G > A	1/68	218.55 (1.8–58.4)	**0.0072**	0.1082	218.55 (1.8–58.4)	**0.0072**	0.1080	210.69 (1.8–56.5)	**0.0074**	0.1110	17.50 (0.6–19.2)	0.0855	1.0000
*NOTCH2*	c.4688G > A	1/68	1078.80 (2.7–159.4)	**0.0017**	**0.0252**	384.47 (0.5–328.5)	**0.0078**	0.1170	972.38 (2.6–152.3)	**0.0018**	**0.0270**	402.07 (0.5–334.9)	**0.0074**	0.1110

a *Odd ratios were calculated in the 95% confidence interval*.

b *p-value adjusted for multiple testing by the total amount of variants found for each gene following Bonferroni approach*.

*The bold values denote statistical significance at the p < 0.05 level*.

### Phenotypes of Patients With a Genetic Variant

The mean age of onset in patients with a novel/rare variant was 53 ± 11.7 years, which showed a tendency of early-onset disease rather than the patients without a genetic variant, but this was not significantly different (vs. 58.4 ± 11.1 years, *p* = 0.133, Table 3). Also, no significant differences were observed between the two groups in terms of sex, bilaterality, the type of MD, and hearing threshold at diagnosis (Table 3). Three patients with variants in two or more genes did not have distinct clinical features compared to those without variants.

**Table 3:** Phenotypic differences between patients with and without a genetic variant.

	**Genetic variant (+), *n* = 12**	**Genetic variant (–), *n* = 56**	***p*-value**
Sex, female, *n* (%)	7 (58)	31 (55)	0.851
Age of onset, mean ± SD	53 ± 11.7	58.4 ± 11.1	0.133
Bilaterality, *n* (%)	0 (0)	5 (9)	0.576
Classic MD, *n* (%)	10 (83)	40 (71)	0.490
Hearing threshold at diagnosis (dB)^a^	49 ± 31.1	49.6 ± 24.3	0.941

a * Hearing threshold was defined as four pure tone average 0.5, 1, 2, and 3 kHz according to the AAO-HNS criteria*.

## Discussion

To the best of our knowledge, this is the first study on extensive genetic screening with regard to putative candidate MD genes. By using targeted NGS, we identified 15 rare heterozygous variants in 11 candidate MD genes, which some of them had the pathogenic potential by *in silico* prediction or protein structural modeling. Our results highlight the genetic landscape of MD.

Over the last 20 years, many candidate genes have been proposed for MD based on the so-called “common disease-common variants” paradigm that prevailed in complex disease genetic studies (8–10). It was thought that the common variants may lead to genetic susceptibility to complex polygenic disease (43). By this approach, some SNPs associated with MD have been identified in the genes related to the inflammation or regulating the ionic composition and water transport of the inner ear (11–35). However, most of the SNPs had small effect size with an odds ratio <2, and several variants were located in the non-coding regions of the genome. In addition, none of them would be replicated among a different ethnic group (26, 44, 45). These suggest that a common genetic variant cannot explain the entire heritability of MD and another investigation may be needed (46).

The MD has strong familial aggregation, and most of these families show an autosomal dominant mode of inheritance with incomplete penetrance (3–5). Thus, the optimal methods identifying novel genes may be a NGS technique or a massive parallel sequencing targeting for familial MD. Recent advances in molecular diagnostics have made it possible to explore the entire genome of individuals. By using WES, a Spanish group has identified novel or rare variants in the *FAM136A, DTNA, PRKCB, DPT*, and *SEMA3D* in several families with MD (36–38). These proteins were expressed in the neurosensorial epithelium of the crista ampullaris of the rat by immunohistochemistry. This suggests that they may play a significant role in the formation or maintaining of inner ear structures, and the identified rare variants are expected to contribute to the development of EH by the altered protein functions. In this study, we identified some rare variants of *FAM136A, DTNA*, and *DPT* genes in sporadic cases. Especially, one variant in *DTNA* (c.2094G > A) had highly pathogenic potential by truncating the protein. The *DTNA* encodes α-dystrobrevin (DB), a structural protein of the dystrophin-associated protein complex (36). The absence of α-DB resulted in abnormal brain capillary permeability, progressively escalating brain edema in the mouse model (47). Also, it was expressed in the vestibular system at early stages of development in mice, suggesting a relevant role in the maturation of the vestibular system (48). Although their pathogenicity should be confirmed by functional study, the rare variants in familial MD genes may have large effects on phenotype by impairing protein function, and gene-environment interactions may be strongest for rare alleles (49–51).

In addition, we detected some rare variants in the immune-associated genes including *PTPN22, NFKB1, TLR2*, and *CXCL10*. There have been growing evidences on the role of autoimmunity and immunological mechanisms in the development of MD as follows: (1) The increased prevalence of autoimmune disease among MD patients, (2) The elevated levels of autoantibodies and immunocomplexes in MD patients, (3) The association of MD with HLA-types and genetic polymorphisms, and (4) The positive response to steroid (52–54). Recent studies have found that basal levels of proinflammatory cytokines were increased in some patients with MD (55, 56). Gene expression study using mRNA also demonstrated that immune-related genes such as *GSTM1, TMEM176A*, and *TMEM176B* were highly expressed in MD patients compared to normal controls (57). Through our study, the *PTPN22* may be of particular interest to support the immune-associated process in MD. The identified rare variants had a pathogenic potential by truncating protein or inducing structural instability. The *PTPN22* encodes lymphoid tyrosine phosphatase (LYP), which is a strong negative regulator of T cell activation, and is expressed in various immunocytes including T- and B-cells (58). It has been found to be associated with autoimmune diseases such as rheumatoid arthritis, systemic lupus erythematosus, and type 1 diabetes (59–61). A previous study also found significant association between putative functional SNP (rs2476601) and bilateral MD in the Spanish population (31). The *NFKB1* encodes a transcription factor that regulates inflammation and immune responses, and has been linked to a number of inflammatory diseases, such as autoimmune arthritis, asthma, and glomerulonephritis (15). Some allelic variants in *NFKB1* are known to modify the hearing outcome in patients with MD and unilateral SNHL (15). The *TLR2* is a member of the Toll-like receptor family, which plays a fundamental role in pathogen recognition and activation of innate immunity (40). The *CXCL10* encodes interferon gamma-induced protein 10 (IP-10), a small cytokine belonging to the CXC chemokine family (40). IP-10 is an important mediator of the inflammatory response to interferons, and has been reported to contribute to immune mediated apoptosis in the ear, inducing human presbycusis (62). All these things support the possible autoimmune etiology or immunological mechanisms in the development of MD.

For several decades, a vascular theory has been proposed as the mechanism of MD's attacks (63). The classic attacks are characterized by acute loss of vestibular response and low-tone hearing followed by an apparent recovery over hours. These unique characteristics may be explained by the differential sensitivity of inner ear tissues to transient ischemia and the excitotoxic cascade lasting several hours by ischemia/reperfusion injury (63). Most animal models demonstrated that both the endolymphatic hydrops and the impaired perfusion pressure were needed to induce MD's attacks (64, 65). Many studies have also documented an association between MD and presumed vascular disorders including migraine, sickle cell disease, Behcet's disease, and branch retinal artery occlusion (66–71). Among the identified genes in this study, several genes including *MTHFR, NOS3, SLC44A2*, and *NOTCH2* are associated with prothrombotic risk factors and various vascular disorders (72–75). The *MTHFR* encodes methylenetetrahydrofolate reductase which catalyzes the conversion of 5,10-methylenetetrahydrofolate to 5-methyltetrahydrofolate, a co-substrate for homocystein remethylation to methionine (29). Specific polymorphisms in *MTHFR* (rs1801131, rs1801133) have been proposed as predisposing inherited vascular risk factors in the development of MD and sudden SNHL (29, 76, 77). The *NOS3* encodes a nitric oxide synthase 3, which is responsible for the generation of nitric oxide (NO), a vasodilator in the vascular endothelium (13). NOS3 is also located in the inner and outer hair cells, and a polymorphism of *NOS3* (rs1799983) was significantly associated with the risk of sudden SNHL and MD (13). The *SLC44A2* encodes a choline transporter-like protein 2 (CTL2) which plays a role in choline transport or uptake with CTL1 (32). Two polymorphisms in *SLC44A2* (rs2288904, rs9797861) were linked to venous thrombosis, coronary artery disease, and stroke, and rs2288904 was associated with severity of MD (32). Furthermore, *in vivo* binding of anti-SLC44A2 antibody induced hearing loss in mice and guinea pigs (78, 79). Thus, their genetic deficiency may cause the increased permeability of vascular endothelial cells, oxidative stress response, and increased vascular resistance, resulting in thrombosis and disturbance of micro-circulation in the inner ear (32).

Although the diagnostic criteria established by the Classification Committee of the Barany Society may improve the accuracy in clinical diagnosis of MD, the phenotypic heterogeneity is observed and some patients may have co-morbid conditions with MD, such as migraine or autoimmune disorders (39). Recent study suggests that an enrichment of rare variants in hearing loss genes such as *GJB2, SLC26A*, or *USH1G* may contribute to explain these phenotypic heterogeneities (80). Our patients in this study also showed a broad phenotypic spectrum regarding the onset age, clinical subgroups, and hearing threshold at diagnosis, and some had an accumulation of rare variants in two or more MD genes. Although we confined our gene panel to putative candidate genes associated with MD, our results also suggest the additive effect of several rare variants in the variable expressivity of MD phenotype. Alternatively, the burden of copy number variation can help understand phenotypic heterogeneity (81). Further studies may be needed to confirm these theories.

This study has potential limitations. Despite the extensive genetic screening using NGS, more than 80% of our patients did not have any likely pathogenic variant in putative candidate MD genes. This low detection rate may be due to a high proportion of sporadic cases in our study. Or, the unknown genes or additional factors including epigenetic and environmental modification are likely to contribute to the development of MD (46). We also did not perform functional study determining pathogenicity of our variants. Indeed, several variants in our study were predicted to be benign by *in silico* prediction tools, or affect only some isoforms of a gene. Despite the rarity and putative pathogenicity of the variants, establishing the pathogenicity may be difficult without a functional study, especially in sporadic cases. With regard to variants of familial MD gene, we could not determine if they were *de novo* mutations because of parental death. Finally, our panel did not include the *GSTM1* or *histamine H4 receptor* genes, which have recently been suggested as a possible candidate gene of MD (57, 82). All of these should be considered when interpreting our results.

In conclusion, we identified rare variants of putative candidate genes in some of MD patients. The identified genes were related to the formation of inner ear structures, the immune-associated process, or systemic hemostasis derangement, suggesting the multiple genetic predispositions in the development of MD. Since there are still many MD patients without genetic variants, further assessments for the candidate genes will be needed.

## Data Availability Statement

The raw data supporting the conclusions of this manuscript will be made available by the authors, without undue reservation, to any qualified researcher.

## Ethics Statement

All experiments followed the tenets of the Declaration of Helsinki, and informed consents were obtained after the nature and possible consequences of this study had been explained to the participants. This study was approved by the institutional review boards of Pusan National University Yangsan Hospital.

## Author Contributions

EO analyzed and interpreted the data and wrote the manuscript. J-HS and H-SK perfomed the experiment. SC, K-DC, J-KR, and JC analyzed and interpreted the data. SL and CL interpreted the data and revised the manuscript. J-HC contributed to the design and conceptualization of the study, and revised the manuscript.

### Conflict of Interest

The authors declare that the research was conducted in the absence of any commercial or financial relationships that could be construed as a potential conflict of interest.
