# Supplementary material for: Rare Variants of Putative Candidate Genes Associated With Sporadic Meniere's Disease in East Asian Population
Source: Front Neurol. 2020 Jan 22;10:1424. doi: 10.3389/fneur.2019.01424 (PMC6987317; doi:10.3389/fneur.2019.01424)
Supplement: Supplementary file 1 [file Table_1.DOCX]

**Supplementary Table 1.** Genes associated with Menière’s disease and their transcript IDs.

| **Gene symbol** | **Genbank transcript ID** | **Reference** |
| --- | --- | --- |
| Familial MD genes | | |
| *DPT* | NM_001937.4 | [38] |
| *DTNA* | NM_001198938.1 | [36] |
| *FAM136A* | NM_032822.2 | [36] |
| *PRKCB* | NM_002738 | [37] |
| *SEMA3D* | NM_152754.2 | [38] |
| MD-associated genes | | |
| *ADD1* | NM_014189.3 | [11] |
| *AQP4* | NM_001650.4 | [12] |
| *AQP5* | NM_001651 | [12] |
| *CAV1* | NM_001172895.1 | [13] |
| *CCL2* | NM_002982.3 | [40] |
| *CCL5* | NM_002985 | [14] |
| *CD4* | NM_000616.4 | [40] |
| *CHGA* | NM_001275 | [16] |
| *CXCL9* | NM_002416.1 | [40] |
| *CXCL10* | NM_001565.3 | [40] |
| *GPX4* | NM_001039847.2 | [40] |
| *GPX5* | NM_001509 | [40] |
| *HCFC1* | NM_005334 | [17] |
| *HLA-C* | NM_002117 | [18,19] |
| *HLA-DRB1* | NM_002124 | [20-22] |
| *HSPA1A* | NM_005345 | [23] |
| *IL 1A* | NM_000575.3 | [24] |
| *IL-1R1* | NM_000877.3 | [40] |
| *IL-6* | NM_000600.3 | [40] |
| *IL 10* | NM_000572.3 | [40] |
| *IL 18* | NM_001562.3 | [40] |
| *KCNE1* | NM_000219.5 | [25,26] |
| *KCNE3* | NM_005472 | [25,26] |
| *MICA* | NM_001177519 | [27] |
| *MIF-173* | NM_002415 | [28] |
| *MTHFR* | NM_005957.4 | [29] |
| *MYD88* | NM_001172567 | [40] |
| *NFKB1* | NM_003998.3 | [15] |
| *NOS3* | NM_000603.4 | [13] |
| *NOTCH2* | NM_024408.3 | [40] |
| *PARP-1* | NM_001618 | [30] |
| *PTGS2* | NM_000963 | [40] |
| *PTPN22* | NM_015967.5 | [31] |
| *SIK1* | NM_173354.3 | [35] |
| *SLC44A2* | NM_020428.3 | [32] |
| *SLC8A1* | NM_021097.2 | [35] |
| *TLR2* | NM_003264 | [40] |
| *TLR9* | NM_017442.3 | [40] |
| *TLR10* | NM_001017388.2 | [33] |
| *TNF* | NM_000594 | [34] |
